# Health Tract, No. 139

**Published:** 1865

**Authors:** 


ONE of the most general causes of unthrift to farmers, as well as reasons why many persons who retire to the country to spend the evening of their days, after having accumulated a fortune in the city, and soon tire or become dissatisfied, is the unwise grasping for too much land. The farmer wants from the first to secure enough to be a little fortune for each child, by the rise in price. The citizen can not rid himself of ideas about profit and loss; and his mind will run on the fact, that if he gets a good slice of land, it may turn out that he can divide it into town-lots in a few years, and realize an immense per centage; but while he is waiting for a town, a messenger comes to say, "You are wanted"—for the last great account! The young farmer, after working out a little lifetime in trying to pay interest, wakes up some morning to find that he has already paid more for his farm than it is worth, and is owing a considerable amount on it besides; for the "rise" never came! Let the merchant remember that going to the country will kill him all the sooner, if he does not at the same time go to work; that the vexations attendant on a large place, which is equivalent to embarking in a new business, one about which he knows almost nothing, will inevitably produce a disquietude of mind, and at length a general irritation of temper, many fold more injurious to his well-being than if he had remained in business. As much work can be profitably expended on one acre of arable soil as any retired merchant ought to perform in twelve months. And there are farmers, wise beyond their day, who, by expending on one acre the labor which others have diffused over twenty, have saved more money, lived more quietly, enjoyed more happiness, and reveled in more luscious good health. By what follows, it may be seen how a man made money for two successive years, by cultivating one acre of land well; planting potatoes the first year, following them with wheat.

*Dr.*

| | |
|---|---|
| **POTATOES.** | |
| To 12 loads manure, | $10 00 |
| Hauling and spreading same, | 3 00 |
| Plowing in potatoes, | 8 75 |
| 11½ bushels seed, at 90 cents, | 10 35 |
| Hoe-harrowing and hoeing, | 3 25 |
| Digging and putting in cellar, | 24 87½ |
| Hauling to market, (10 miles,) | 6 25 |
| **WHEAT.** | |
| Harrowing, | 1 50 |
| Seeding, | 87½ |
| 1½ bushels seed, at $1.30, | 1 95 |
| Cradling and hauling in, | 2 50 |
| Threshing and cleaning, | 2 50 |
| Hauling to market, (2 miles,) | 75 |
| | $76 05 |

*Cr.*

| | |
|---|---|
| To 218 bushels potatoes, at 97 cents, | $211 46 |
| Tops as manure, | 3 00 |
| 31 bushels wheat, at $1.25, | 38 75 |
| 1 ton straw, | 8 00 |
| Chaff, | 1 00 |
| Cr., | $272 21 |
| Dr., | 76 05 |
| | $186 16 |
| Interest on land, 17 months, | 2 75 |
| | $183 41 |

The land was a good loam, with a light clover sod. The manure was spread on the sod, and plowed down with the potatoes in every third (narrow) furrow. The seed was the common Mercer, planted as early as convenient, and dug ditto; no sign of rot. The wheat was the common blue-stem. The potatoes were plowed out every third furrow, and the ground was plowed regularly, and harrowed down for wheat.

Let all who seek fortune or health in farming remember to purchase no more land than they can pay for, and no more than they can easily cultivate with the force they have; otherwise, irritations, vexations, and disappointments will eat out their health and squander their money.

